# Supplementary material for: Reciprocal best structure hits: using AlphaFold models to discover distant homologues
Source: Bioinform Adv. 2022 Oct 6;2(1):vbac072. doi: 10.1093/bioadv/vbac072 (PMC9666668; doi:10.1093/bioadv/vbac072)
Supplement: vbac072_Supplementary_Data [file vbac072_supplementary_data.pdf]

Supplementary

Table S1: Results overview: Number of detected RBSH, RBH and commonly found closest homologous protein pairs between *H. sapiens* and the four model organisms as well as between *S. cerevisiae* and *S. pombe*.

	H. sapiens - *D. melanogaster*	*H. sapiens* - *C. elegans*	*H. sapiens* - *S. cerevisiae*	*H. sapiens* - *S. pombe*	*S. cerevisiae* - *S. pombe*
RBSH	4,316	3,837	1,921	2,095	2,751
RBH	3,306	2,824	1,245	1,423	1,949
Common	2,706	2,251	979	1,175	1,706

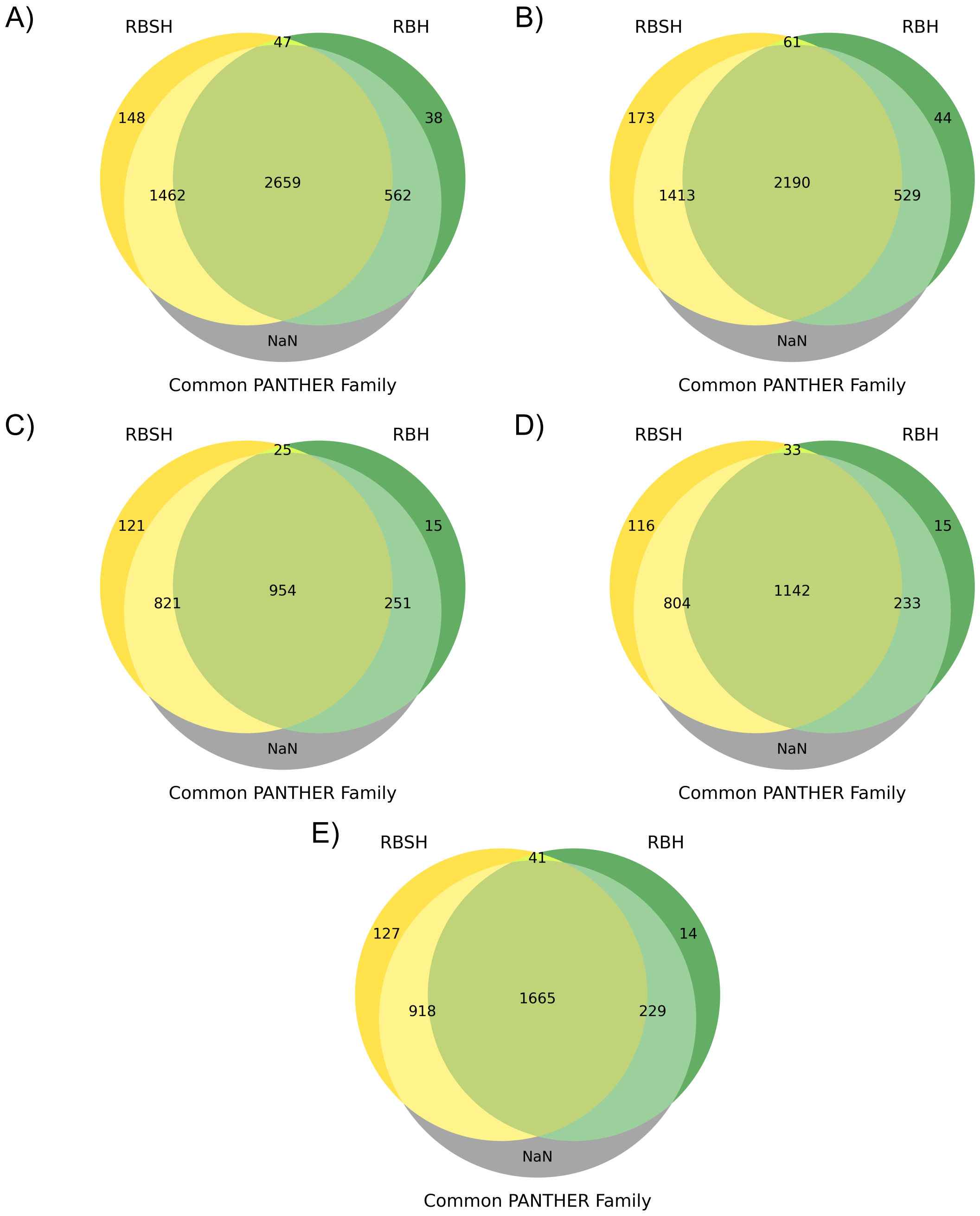

Figure S1: Comparison of RBH and RBSH methods with PANTHER family classification: comparison between RBSH detected with Foldseek and RBH detected with BLASTP for A) *H. sapiens* and *D. melanogaster* B) *H. sapiens* and *C. elegans*, C) *H. sapiens* and *S. cerevisiae*, D) *H. sapiens* and *S. pombe*, and E) *S. cerevisiae* and *S. pombe*. 'Common Panther Family' represents how many of the RBH or RBSH found with the two approaches have a common PANTHER family classification. The number of protein pairs with a common PANTHER Family classification were not counted ('NaN').

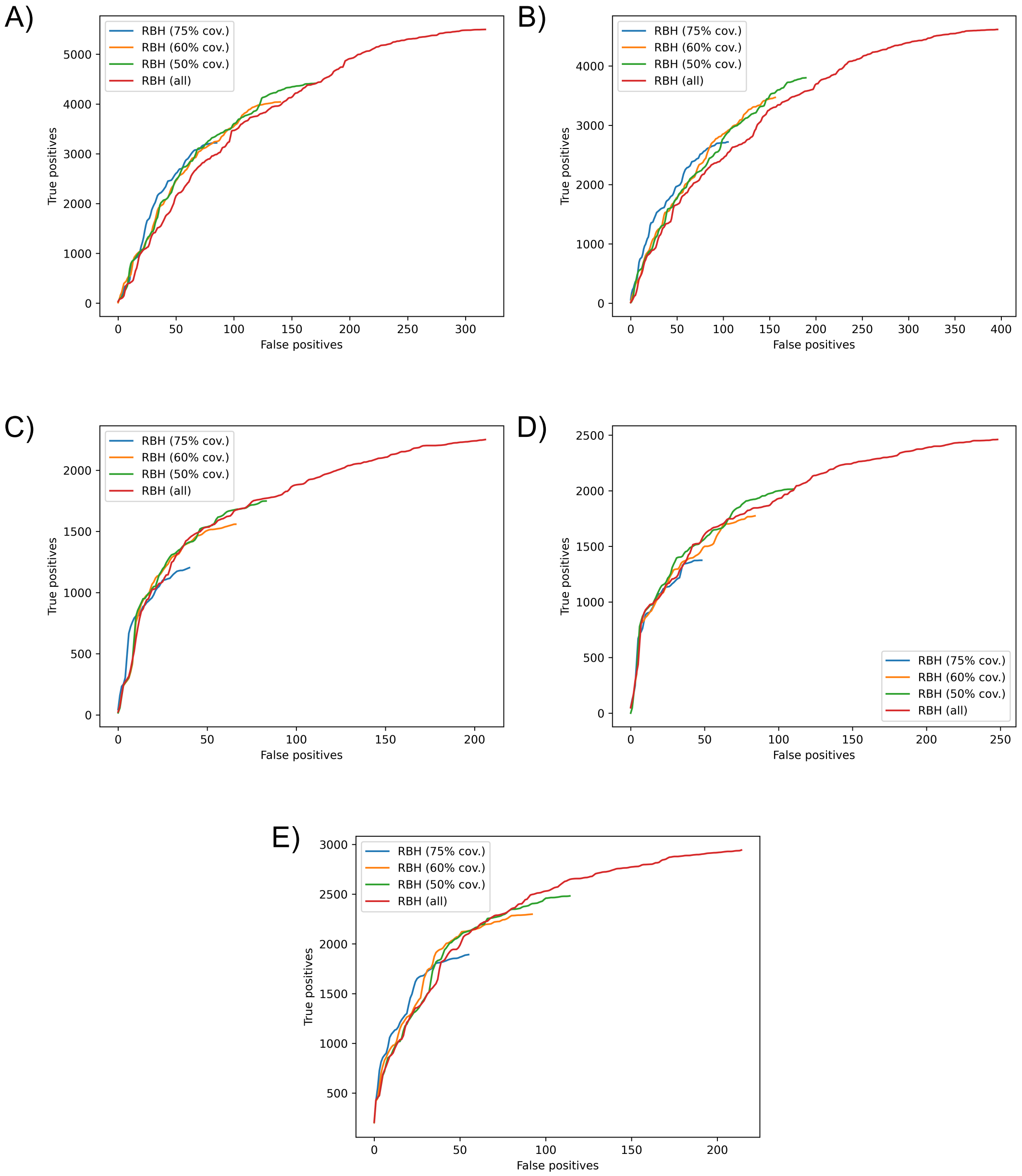

Figure S2: Investigating different coverage thresholds for the RBH approach: Validation of the RBH method testing different coverage thresholds. 'True positives' represents the protein pairs with the same PANTHER family, and 'False positives' represents the protein pairs without the same PANTHER family or without any PANTHER classification. The results are shown for A) *H. sapiens* and *D. melanogaster*, B) *H. sapiens* and *C. elegans*, C) *H. sapiens* and *S. cerevisiae* D) *H. sapiens* and *S. pombe* and E) *S. cerevisiae* and *S. pombe*.

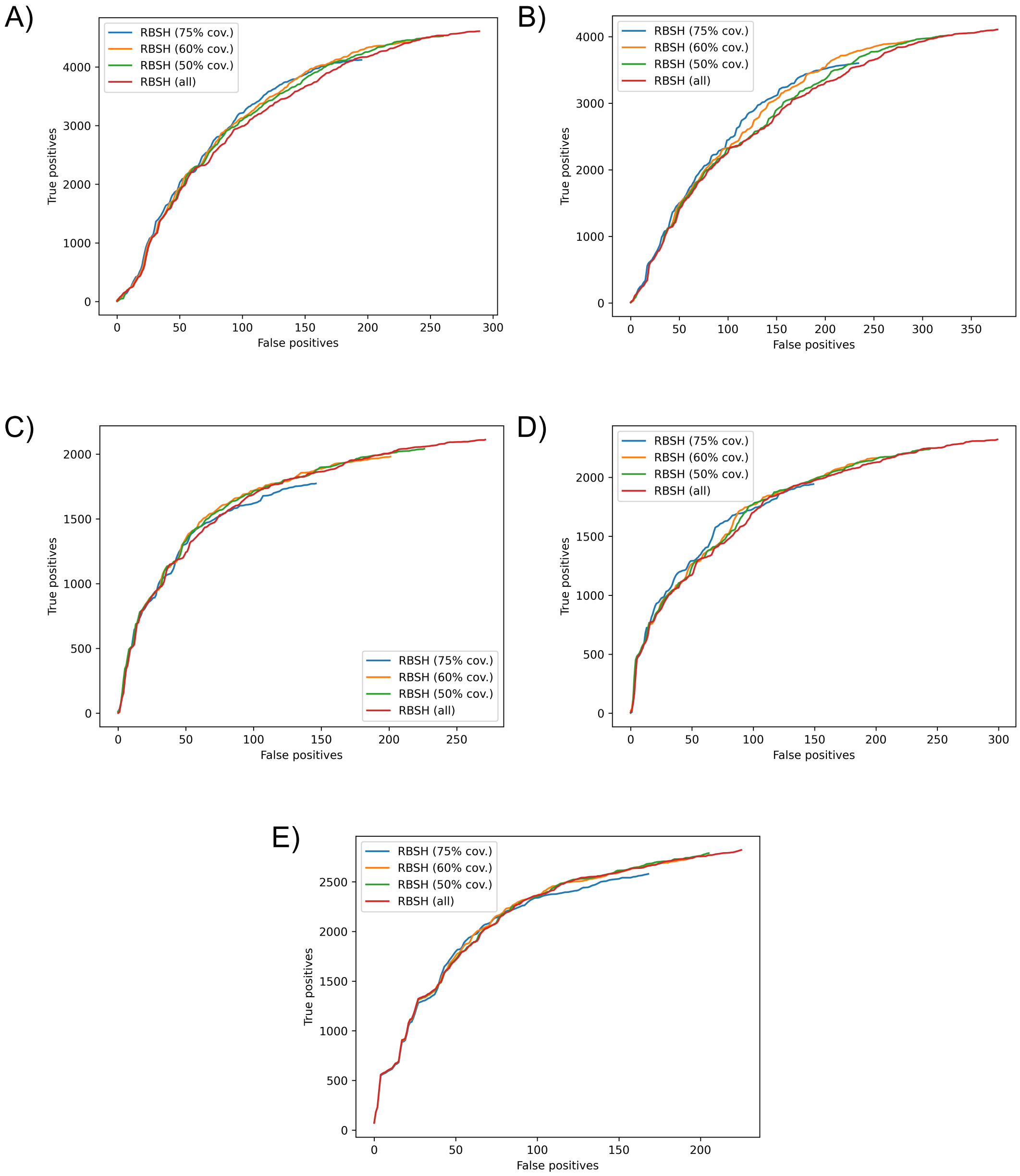

Figure S3: Investigating different coverage thresholds for the RBSH approach: Validation of the structural matching approach testing different coverage thresholds. 'True positives' represents the protein pairs with the same PANTHER family, and 'False positives' represents the protein pairs without the same PANTHER family or without any PANTHER classification. The results are shown for A) *H. sapiens* and *D. melanogaster*, B) *H. sapiens* and *C. elegans*, C) *H. sapiens* and *S. cerevisiae* D) *H. sapiens* and *S. pombe* and E) *S. cerevisiae* and *S. pombe*.

Table S2: Number of homologous proteins found on average: the table lists the number of homologous proteins found on average per query protein representing the different number of choices on average to form a reciprocal best hit (RBH) or reciprocal best structure hit (RBSH). The results distinguish all proteins found in a reciprocal hit (all) and proteins found by both the RBH and RBSH methods (common).

	H. sapiens (p1) - *D. melanogaster* (p2)		*H. sapiens* (p1) - *C. elegans* (p2)		*H. sapiens* (p1) - *S. cerevisiae* (p2)		*H. sapiens* (p1) - *S. pombe* (p2)		*S. cerevisiae* (p1) - *S. pombe* (p2)	
	p1	p2	p1	p2	p1	p2	p1	p2	p1	p2
RBH all	4.77	5.97	4.44	5.34	2.77	3.99	2.52	3.83	2.09	2.23
RBH common	2.42	2.82	2.53	2.99	1.99	2.83	2.03	2.93	1.78	1.85
RBSH all	9.01	12.72	18.16	20.4	6.26	10.72	6.0	10.85	5.94	6.36
RBSH common	5.28	6.71	5.9	6.89	3.51	5.88	3.93	6.83	4.9	5.11

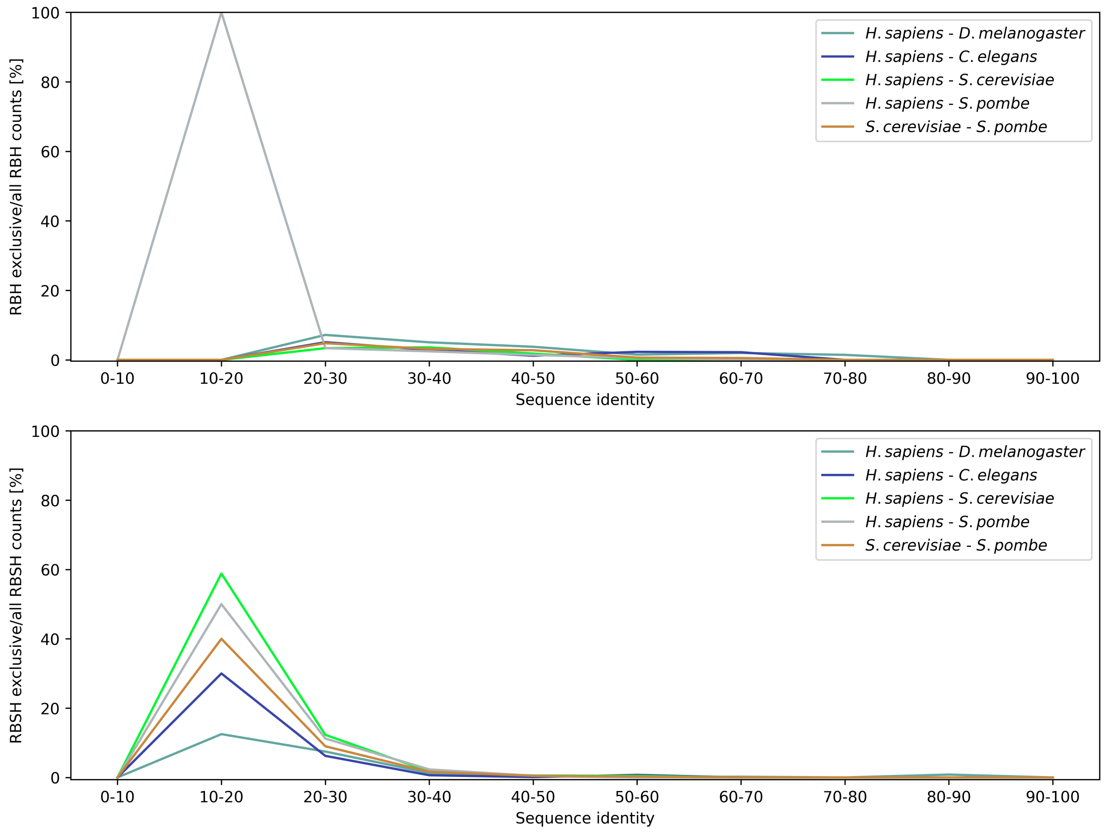

Figure S4: Sequence similarity of protein pairs exclusively found by the RBH or RBSH method: Number of protein matches found exclusively by one approach normalised by the total number of matches found by this approach, plotted against the sequence identity calculated between the protein pairs. Analysis results for the different model organisms are differentiated between the RBH approach (plot above) and RBSH approach (plot below).

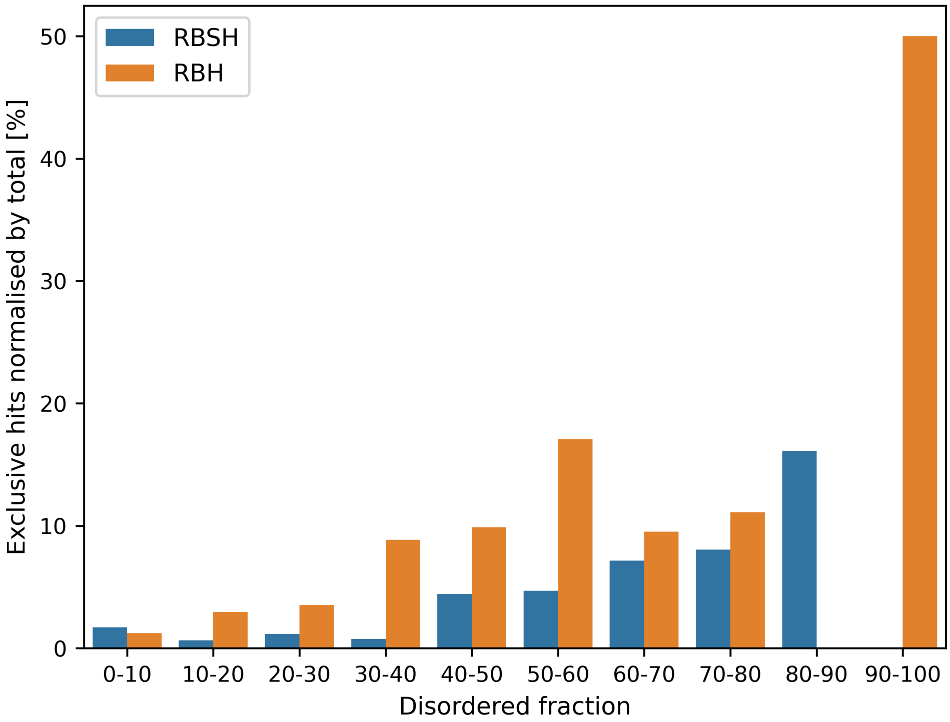

Figure S5: Percentage of protein matches exclusively found by the RBSH or RBH method per disordered protein fraction: This plot shows the results of the protein matches between *H. sapiens* and *D. melanogaster*, exemplary for the results with the other model organisms.

Table S3: Precision score comparison: Comparison of RBSH approach results with original structure models and only high confidence regions of the structure models (>50 pLDDT score). Precision scores calculated based on PANTHER classification.

	H. sapiens - D. melanogaster	*H. sapiens - C. elegans*	*H. sapiens - S. cerevisiae*	*H. sapiens - S. pombe*	*S. cerevisiae - S. pombe*
RBH	0.974	0.969	0.973	0.968	0.973
RBSH (75% cov.)	0.955	0.939	0.924	0.929	0.939
RBSH (no cov. thres.)	0.941	0.916	0.886	0.886	0.926
RBSH confidence score >50 (75% cov.)	0.955	0.932	0.911	0.917	0.938
RBSH confidence score >50 (No cov. thres.)	0.942	0.91	0.873	0.881	0.926

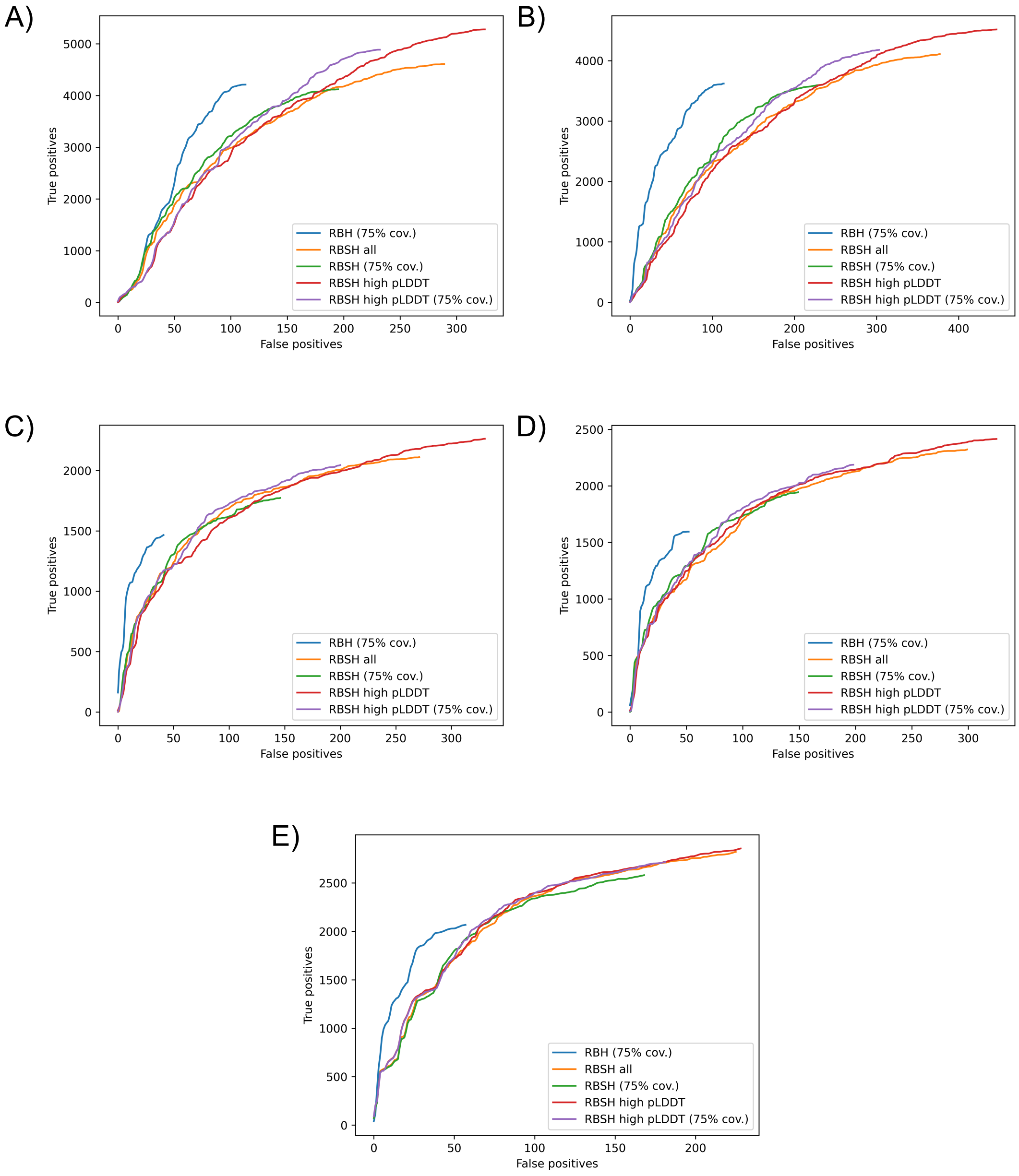

Figure S6: Validation plots with results of the RBSH approach using pdb files without low confidence regions: validation plots as shown in figure S2 plus the validation results of RBSH methods using the adapted pdb files. These pdb files do not compromise regions with an AlphaFold confidence prediction below 50 (pLDDT score). 'True positives' represents the protein pairs with the same PANTHER family, and 'False positives' represents the protein pairs without the same PANTHER family or without any PANTHER classification. The results are shown for A) *H. sapiens* and *D. melanogaster*, B) *H. sapiens* and *C. elegans* C) *H. sapiens* and *S. cerevisiae* D) *H. sapiens* and *S. pombe* and E) *S. cerevisiae* and *S. pombe*.

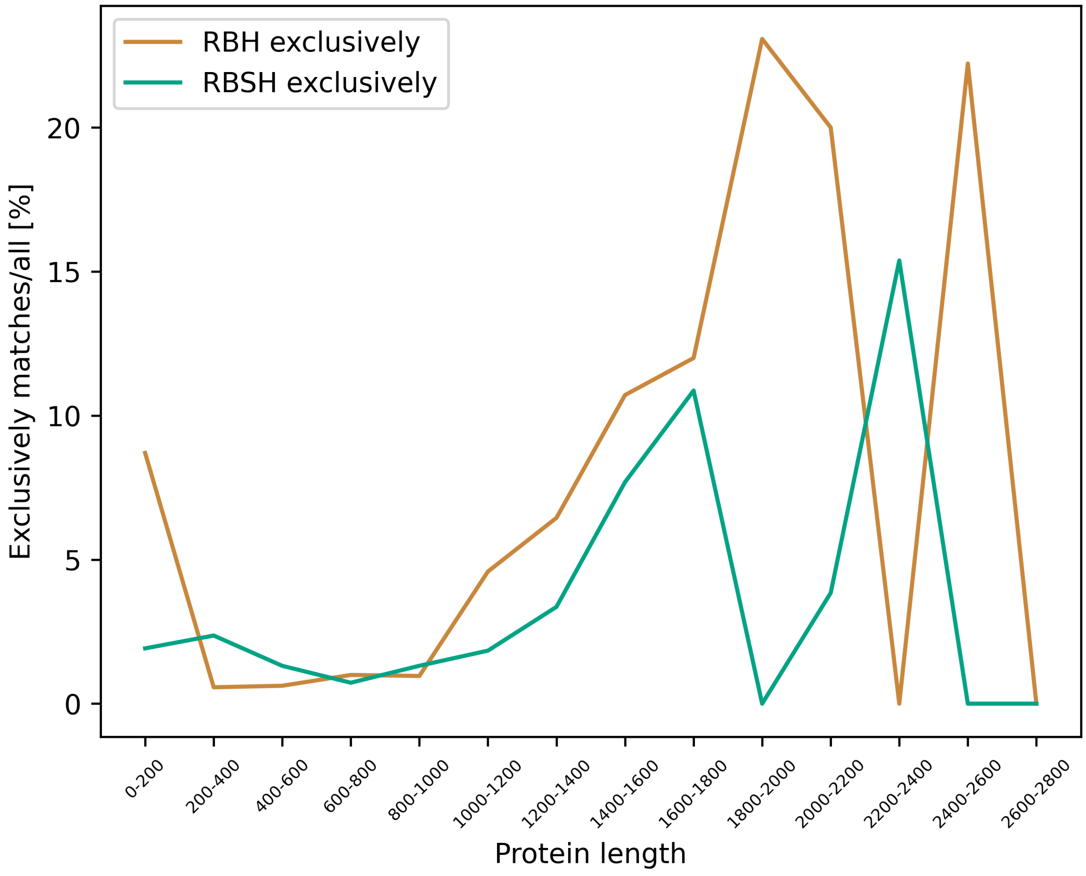

Figure S7: Percentage of protein matches exclusively found by the RBSH or RBH method per protein length: this plot shows the results for the protein matches between *H. sapiens* and *D. melanogaster*, exemplary for the results with the other model organisms.

Table S4: Comparison between the best structure hit and reciprocal best structure hit: The precision score between the RBSH and best structure hits are compared for the structural matching approach. Precision scores are calculated based on the PANTHER classification. For the best structure hits, the precision is calculated for the best hits selected in both directions, meaning both organisms being once the query proteome, and the average score is listed here.

	RBSH	Best structure hit (average)
H. sapiens: *D. melanogaster*	0.955	0.707
H. sapiens: *C. elegans*	0.939	0.613
H. sapiens: *S. cerevisiae*	0.924	0.583
H. sapiens: *S. pombe*	0.929	0.605
S. cerevisiae: *S. pombe*	0.939	0.844

Table S5: AlphaFold RBSH predictions added to PomBase curated orthologs: Detected between *S. pombe* and *H. sapiens*.

S. pombe			*H. sapiens*	
Systematic identifier	Gene symbol	UniProt	HGNC appoved name	UniProt
SPAC12B10.02c	pho86	Q10436	NAT8	Q9UHE5
SPCC1682.03c	mug174	O74434	COIL	P38432
SPAP27G11.02	mgr3	Q9P7N6	TTC19	Q6DKK2
SPCC1753.02c	git3	O94744	GPR101	Q96P66
SPAC1952.09c	ach1	Q9UUJ9	OXCT1	P55809
SPCC1393.14	ten1	P0C5Y7	TEN1	Q86WV5
SPAC1952.08c		Q9UUK0	CREG1	O75629
SPAC2F3.01	imt1	O14084	A4GALT	Q9NPC4
SPAC922.05c		Q9URX1	UNC93A	Q86WB7
SPBC21.02	rtc5	O94644	MEAK7	Q6P9B6
SPBC216.03		Q9Y7K0	BLVRB	P30043

Table S6: AlphaFold RBSH predictions added to PomBase curated orthologs: Detected between *S. sapiens* and *S. cerevisiae*

S. pombe			*S. cerevisiae*	
Systematic identifier	Gene symbol	UniProt	SGD gene name & locus ID	UniProt
SPAC1B3.10c		O13875	HRD3/YLR207W	Q05787
SPBC29A3.17	gef3	O59679	FUS2/YMR232W	Q05670
SPAC13G6.08	fzr2	Q09786	AMA1/YGR225W	P50082
SPCC306.05c	ins1	Q9Y7R5	NSG1/YHR133C	P38837
SPBC19G7.08c	art1	O42956	RGL1/YPL066W	Q12194
SPBC1289.06c	ppr8	O94615	RMD9/YGL107C	P53140
SPBC16G5.16		O60130	AGS1/YIL130W	P40467
SPCP25A2.03	tho1	Q9URT2	HPR1/YDR138W	P17629
SPAC3G9.17	new8	G2TRL9	PPT2/YPL148C	Q12036
SPCC126.08c		O94401	UIP5/YKR044W	P36137
SPAC1002.07c		P79081	HPA2/YPR193C	Q06592
SPCC1672.10	mis16	O94244	HAT2/YEL056W	P39984

SPAC1952.12c	csn71	Q9UUJ7	CSN9/YDR179C	Q03981
SPAC3C7.04		O14130	PUT3/YKL015W	P25502
SPAC8E11.04c		O42881	YLR118C	Q12354
SPBP19A11.06	lid2	Q9HDV4	ECM5/YMR176W	Q03214
SPAC1399.05c	toe1	Q9HE16	URC2/YDR520C	Q04411
SPBC19C2.04c	ubp11	Q9UUD6	UBP16/YPL072W	Q02863
SPAC323.03c		Q9UT96	PEX8/YGR077C	P53248
SPCC1753.05	rsm1	O94506	PML39/YML107C	Q03760
SPAC12G12.12	gms2	Q09875	YPL264C	Q08980
SPAC17C9.16c	mfs1	Q10487	FLR1/YBR008C	P38124
SPAC23E2.01	fep1	Q10134	GZF3/YJL110C	P42944
SPBC16E9.19	pac3	Q9C0X2	IRC25/YLR021W	Q07951
SPCC24B10.16c	pac4	Q9P7J0	POC4/YPL144W	Q12245
SPBC8D2.07c	sfc9	O13650	TFC8/YPL007C	Q12308
SPBC83.10	emc7	O94694	SOP4/YJL192C	P39543
SPBC16C6.01c		O42925	RKM3/YBR03W	P38222
SPAC31G5.09c	spk1	P27638	KSS1/YGR040W	P14681
SPBC16E9.09c	erp5	O14324	ERP1/YAR002C-A	Q05359
SPBC215.05	gpd1	P21696	GPD2/YOL059W	P41911
SPBC609.01		O94525	SSD1/YDR293C	P24276
SPCC757.09c	rnc1	O74919	YBL032W/HEK2	P38199
SPAC24B11.12c	dnf2	Q09891	DNF1/YER166W	P32660
SPBC16A3.17c		O42922	VBA5/YKR105C	P36172
SPBC27B12.12c		O13657	ALR2/YFL050C	P43553
SPBC29A10.11c	vps902	O94388	MUK1/YPL070W	Q02866
SPAC19D5.02c	pex22	Q1K9B6	EMC10/YDR056C	Q12025
SPCC290.04	ams2	Q9URT4	YMR179W/SPT21	P35209
